# Prevalence of the mitochondrial 1555 A>G and 1494 C>T mutations in a community-dwelling population in Japan

**DOI:** 10.1038/s41439-020-00115-9

**Published:** 2020-09-18

**Authors:** Yasunori Maeda, Akira Sasaki, Shuya Kasai, Shinichi Goto, Shin-ya Nishio, Kaori Sawada, Itoyo Tokuda, Ken Itoh, Shin-ichi Usami, Atsushi Matsubara

**Affiliations:** 1 Department of Otorhinolaryngology, Hirosaki University Graduate School of Medicine, Hirosaki, Japan; 2 Department of Stress Response Science, Center for Advanced Medical Research, Hirosaki University Graduate School of Medicine, Hirosaki, Japan; 3 Department of Otorhinolaryngology, Shinshu University School of Medicine, Matsumoto, Japan; 4 Department of Hearing Implant Sciences, Shinshu University School of Medicine, Matsumoto, Japan; 5 Department of Social Medicine, Hirosaki University School of Medicine, Hirosaki, Japan

**Keywords:** Mitochondrial genome, Mutation, Risk factors

## Abstract

Single nucleotide polymorphisms in mitochondrial DNA, such as mitochondrial 1555 A>G (m.1555 A>G) and mitochondrial 1494 C>T (m.1494 C>T), are known to be causative mutations of nonsyndromic hearing loss following exposure to aminoglycoside antibiotics. The prevalence of the m.1555 A>G and m.1494 C>T mutations has not been reported for the general population in Japan. The purpose of this study was to investigate the prevalence of m.1555 A>G and m.1494 C>T mutations in a community-dwelling population in Japan in order to prevent aminoglycoside-induced hearing loss. We recruited participants older than 20 years of age to the Iwaki Health Promotion Project in 2014, 2015, and 2016, resulting in the recruitment of 1,683 participants. For each participant, we performed a hearing test and a genetic test for the m.1555 A>G and m.1494 C>T mutations using the TaqMan genotyping method. The m.1555 A>G mutation was detected in only 1 of the 1,683 participants (0.06%). This carrier of the m.1555 A>G mutation was a 69-year-old male with bilateral, symmetric, and high-frequency hearing loss. We provided genetic counseling and distributed a drug card advising him to avoid the administration of aminoglycoside antibiotics. In contrast, the m.1494 C>T mutation was not detected in this study population.

## Hearing loss: mitochondrial mutations and antibiotic use

Researchers in Japan have measured the prevalence of two hearing loss mutations associated with antibiotic use in the general population. Two mutations in mitochondrial DNA are known to cause hearing loss upon exposure to aminoglycoside antibiotics such as gentamicin. One mutation is found in 3% of Japanese hearing-impaired outpatients, but the frequency in the general population is unknown. A team led by Akira Sasaki of Hirosaki University carried out genetic and hearing tests in 1,683 community-dwelling subjects. They found one person who had one of the mutations. Although this subject had no known history of aminoglycoside antibiotic injection, he exhibited hearing loss at high frequencies. He was provided with genetic counseling and a drug use warning card. The second mutation was not found in the study population.

## Introduction

The World Health Organization (WHO) estimated that over 5% of the world’s population was affected by hearing loss^1^. Hearing loss is associated with an array of problems, such as poor quality of life and negative outcomes related to socialization, independence, interpersonal relationships, and communication^2^. Hearing loss has also been reported to be a significant risk factor for dementia^3^. In addition, hearing loss is associated with additional costs, including health sector costs, the provision of educational support, the loss of productivity, and societal costs, worldwide^1^. Thus, hearing loss may result in a significant burden for both individuals and society.

Previous studies have shown that hearing loss is a common sensory disorder, affecting 1 in 1000 newborns. A total of 54–68% of hearing loss cases have been estimated to have genetic origins or to be associated with genetic predispositions^4^. Causative mitochondrial DNA mutations have been found in approximately 5% of patients with postlingual, nonsyndromic hearing loss^5^. Among the identified mitochondrial mutations, the mitochondrial 1555 A>G (m.1555 A>G) and mitochondrial 1494 C>T (m.1494 C>T) mutations have frequently been identified in patients with aminoglycoside-induced and late-onset, nonsyndromic hearing loss^6,7^.

Many reports have described patients with hearing loss who carry these gene mutations. The m.1555 A>G mutation was reported to occur in approximately 3% of Japanese hearing-impaired outpatients^6^. However, no studies have reported the prevalence of the m.1555 A>G and m.1494 C>T mutations in the general population in Japan. It is well known that in carriers of the m.1555 A>G and m.1494 C>T mutations, hearing loss can progress if aminoglycoside antibiotics are administered. Aminoglycoside antibiotics are still being used in many countries, especially for patients with tuberculosis or methicillin-resistant *Staphylococcus aureus* infection and Neonatal Intensive Care Unit (NICU) babies^8,9^.

However, aminoglycoside-induced hearing loss can be prevented by avoiding the administration of aminoglycoside antibiotics. Therefore, it is very important to investigate the population potentially at high risk for aminoglycoside antibiotic-induced hearing loss.

The purpose of this study was to investigate the prevalence of the m.1555 A>G and m.1494 C>T mutations in a community-dwelling population in Japan in order to prevent aminoglycoside-induced hearing loss.

## Materials and methods

### Subjects

The Iwaki Health Promotion Project is an annual, large-scale, epidemiological survey performed in the Iwaki district of Hirosaki city, Japan^10^. We invited all residents older than 20 years of age who live in this district to participate in this project. The data collected for this project in 2014, 2015, and 2016 were available for this study.

Data collection and genetic analysis for the present study and the Iwaki Health Promotion Project were approved by the Ethics Committee of Hirosaki University School of Medicine (Authorization numbers: 2014-014, 2014-377, 2016-028), and all subjects provided written informed consent before participating in the project.

### Hearing test

Hearing tests were performed for all study participants. Air-conduction, pure-tone thresholds at octave intervals of 0.125, 0.25, 0.5, 1, 2, 4, and 8 kHz were measured by trained examiners using a diagnostic audiometer (AA-73A; Rion, Tokyo, Japan).

### Validation study of the TaqMan genotyping method

To confirm the accuracy of the TaqMan genotyping method used in this study, we performed a validation study by using positive and negative control DNA samples from our previous cohort^11–13^. For the m.1555 A>G mutation, we performed a validation study by using 24 positive control and 24 negative control samples, and for the m.1494 mutation, we performed a validation study with one positive control sample and 24 negative control samples. All TaqMan genotyping results were consistent with the PCR-RFLP, Invader assay, and Sanger sequencing results obtained in our previous studies^11–13^.

### Genetic test

We obtained blood samples from all participants. The presence of the m.1555 A>G and m.1494 C>T mutations was analyzed using the TaqMan genotyping method. Genomic DNA was extracted from 200 µL of a whole-blood specimen using the QIAamp 96 Blood Kit (QIAGEN, Hamburg, Germany). Then, 0.2 μM m.1494T probe, 0.2 μM m.1494C probe, 0.5μM m.1494CT forward primer and 0.5 μM m.1494CT reverse primer or 0.2 μM m.1555G probe, 0.2 μM m.1555A probe, 0.5 μM m.1555AG forward primer and 0.5 μM m.1555AG reverse primer mixed in SsoAdvanced Supermix (Bio-Rad, Hercules, CA, USA) were combined with genomic DNA and analyzed according to the manufacturer’s instructions. Primers and probes were obtained from Eurofins Genomics (Tokyo, Japan), and the sequences are presented in Table 1. A volume of 1 µL of genomic DNA was mixed with 19 µL of premix as a template (3.52–168.9 ng/reaction). Real-time polymerase chain reaction (PCR) was performed using CFX Manager 3.1 software (Bio-Rad) as follows: hot start at 95 °C for 3 min, heat denaturation at 98 °C for 10 s, and extension reaction at 63 °C for 1 min for 40 cycles using CFX 96 or CFX 384 at the 10 µL scale. Fluorescence intensity measured in 30 cycles was used for genotype discrimination.

**Table 1:** List of probe and primer sequences used in this study.

Target	Sequence
Mt1555G probe	5′-FAM-ATG TTA CGA CTT CCT CC-MGBEQ-3′
Mt1555A probe	5′-HEX-ATG TTA CGA CTT TCT CC-MGBEQ-3′
Mt1555 A>G forward primer	5′-GGT CGA AGG TGG ATT TAG CAG TA-3′
Mt1555 A>G reverse primer	5′-AGT GTA AGT TGG GTG CTT TGT GTT AA-3′
Mt1494T probe	5′-FAM-CCG TCA CTC TCC TCA-MGBEQ-3′
Mt1494C probe	5′-HEX-CGT CAC CCT CCT CA -MGBEQ-3′
Mt1494 C>T forward primer	5′-GCC CTG AAG CGC GTA CAC-3′
Mt1494 C>T reverse primer	5′-TCC AGT ACA CTT ACC ATG TTA CGA CTT-3′

## Results

**Fig. 1:**
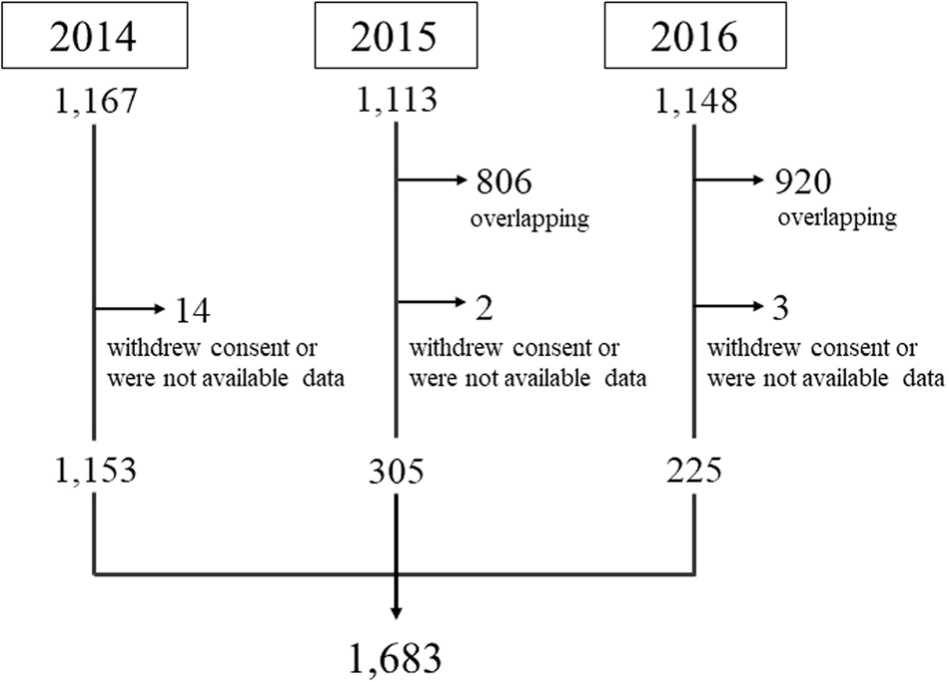
Flow chart illustrating the selection of subjects. A total of 1,167, 1,113, and 1,148 individuals participated in 2014, 2015, and 2016, respectively. We excluded overlapping participants, participants with unavailable data, and participants who did not provide consent for genetic testing. We performed genetic testing for a total of 1,683 subjects.

Approximately 9,500 residents more than 20 years old live in Iwaki district, and 1,167, 1,113, and 1,148 residents participated in this project in 2014, 2015, and 2016, respectively. We excluded overlapping participants, participants without available data, and participants who did not consent to genetic testing. We performed genetic testing for a total of 1,683 subjects (Fig. 1), including 651 males and 1,032 females and ranging from 20 to 91 years of age, with an average age of 53 years.

**Fig. 2:**
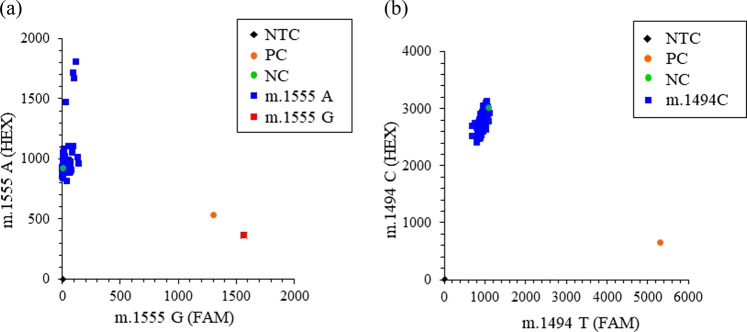
Genetic testing for the mitochondrial DNA SNPs m.1555 A>G and m.1494 C>T. **a** Genetic analysis of m.1555 A>G was performed using the TaqMan genotyping method. Representative data, including data for 1 patient with the m.1555 A>G mutation (red square) and participants with the m.1555 A genotype (blue squares), are shown as two-dimensional plots of m.1555 G amplification (FAM probe signal as the X-axis) and m.1555 A amplification (HEX probe signal as the Y-axis). **b** Representative data for the m.1494 C>T analysis are shown as a plot of m.1494 T amplification (FAM probe signal as the X-axis) and m.1494 C amplification (HEX probe signal as the Y-axis). All participants in this study were genotyped as m.1494 C (blue squares), and the m.1494 C>T mutation was not observed. The black diamond indicates the no-template control (NTC). The orange circle indicates the positive control (PC) [m.1555 A>G in (**a**) and m.1494 C>T in (**b**)]. The green circle indicates the negative control (NC) [m.1555 A in (**a**) and m.1494C in (**b**)].

A genetic test evaluating the presence of the m.1555 A>G and m.1494 C>T mutations was performed using the TaqMan genotyping method (Fig. 2). To detect the m.1555 A>G mutation, a HEX-conjugated probe for m.1555 A and a FAM-conjugated probe for m.1555 G were mixed, and the fluorescence intensity of each was analyzed by real-time PCR. The majority of participants were genotyped as m.1555 A, and only one subject was genotyped as m.1555 A>G (Fig. 2a). In contrast, m.1494 C>T was not observed among any of the subjects in this study (Fig. 2b).

**Fig. 3:**
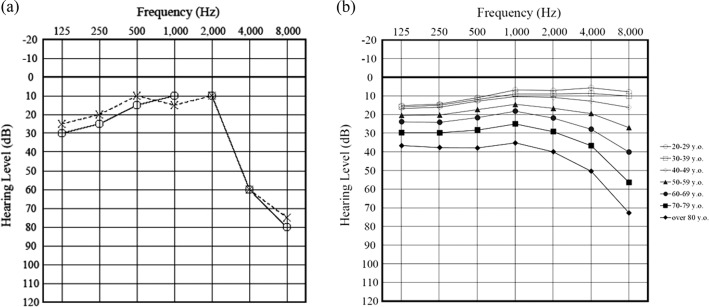
Audiogram for the m.1555 A>G carrier in this study and generation-wise average audiograms for the subjects in this study. **a** The audiogram pattern of the m.1555 A>G carrier showed bilateral, symmetric, and high-frequency (4 and 8 kHz) hearing loss. The right and left audiograms are indicated as circles on a solid line and crosses on a dashed line, respectively. **b** The generation-wise average audiograms for the subjects in this study. The white circle indicates 20–29 years old. The white square indicates 30–39 years old. The white diamond indicates 40–49 years old. The black triangle indicates 50–59 years old. The black circle indicates 60–69 years old. The black square indicates 70–79 years old. The black diamond indicates more than 80 years old.

The subject with the m.1555 A>G mutation in this study was a 69-year-old male. He was diagnosed with hearing loss at a medical check-up when he was 15 years old. Although he had no known history of aminoglycoside antibiotic injection, he described working at a job with noise exposure. The audiogram pattern of this subject showed bilateral, symmetric, and high-frequency hearing loss (Fig. 3a). He has a family history of hearing loss; i.e., his elderly sister suffered from hearing loss at a young age. She was also thought to be an m.1555 A>G carrier. She has two daughters and two granddaughters who have not developed hearing loss. We counseled this individual regarding the risks associated with the use of aminoglycoside antibiotics and provided him with a warning card regarding the administration of these medicines^14^. We also explained to him that the m.1555 A>G mutation is maternally inherited and that his sister and her offspring could have the same gene mutation.

## Discussion

Approximately 50% of childhood hearing loss is estimated to be associated with genetic contributions, and the prevalence of the m.1555 A>G mutation is 1%^4^. Furthermore, the prevalence of the m.1555 A>G mutation has been reported to be 0.42–17% in individuals with hearing loss (Table 2)^6,15–26^. The prevalence of the m.1555 A>G mutation has been reported to be 0.08–0.7% among the general population (Table 2)^27–36^. In this epidemiological study, the m.1555 A>G mutation was identified in 1 out of 1,683 participants from the general population of the Iwaki district (0.06%), which is slightly lower than the frequency in previous reports. However, this estimate is important as a nonbiased frequency in a certain area where the flow of people is low. Recently, SNP information based on 4,700 healthy volunteers has become available, and the frequency of the m.1555 A>G mutation is found to be 0.15%, which is consistent with our frequency　(https://jmorp.megabank.tohoku.ac.jp/202001/).

**Table 2:** Prevalence of m.1555 A>G mutation in individuals with hearing loss and the general population.

Population	Prevalence	Reference
Japan	3.45% (11/319) of outpatients with HL	^6^
Indonesia	5.3% (4/75) of deafness patients	^15^
Greece	0.42% (2/478) of patients with NSHL	^16^
China	3.96% (65/1,642) of individuals with NSHL	^17^
	3.96% (69/1,742) of individuals with NSHL	^18^
	1.6% (7/434) of individuals with NSHL	^19^
	7.5% (33/440) of individuals with HL	^20^
	5.93% (39/658) of individuals with NSHL	^21^
	6.03% (28/464) of individuals with NSHL	^22^
	3.87% (6/155) of individuals with HL	^23^
	10.17% (6/59) of individuals with NSHL	^24^
	1.92% (3/156) of individuals with NSHL	^25^
Spain	17% (9/54) of deafness patients	^26^
United States	0.2% (3/1,473) of general population	^27^
China	0.7% (6/865) of newborns	^28^
	0.16% (16/10,043) of neonates	^29^
	0.08% (1/1,181) of newborns	^30^
	0.17% (101/58,397) of neonates	^31^
Europe	0.19% (18/9,371) of children in ALSPAC birth cohort	^32^
Australia	0.21% (6/2,856) of general population > age 49	^33^
Germany	0.2% (12/7,056) of newborns	^34^
South Africa	0.5% (1/204) of general population	^35^
Taiwan	0.1% (1/1,017) of newborns	^36^
Japan	0.06% (1/1,683) of general population	This study

*HL* hearing loss, *NSHL* nonsyndromic hearing loss, *ALSPAC* Avon Longitudinal Study of Patients and Children

The m.1494 C>T mutation was not observed in any participants in this epidemiological study. The prevalence of the m.1494 C>T mutation has been reported to be 0.18–0.7% among individuals with hearing loss^13,17,20,21,25,37^ and 0.01–0.25% among the general population (Table 3)^27,29–31^. Previous studies reported that the prevalence of the m.1494 C>T mutation was very low, which agreed with the results of this study, in which the m.1494 C>T mutation was not observed among the 1,683 subjects.

**Table 3:** Prevalence of m.1494 C>T mutation in individuals with hearing loss and the general population.

Population	Prevalence	Reference
Japan	0.7% (1/140) of individuals with HL	^13^
China	0.18% (3/1,642) of individuals with NSHL	^17^
	0.45% (2/440) of individuals with HL	^20^
	0.61% (4/658) of individuals with NSHL	^21^
	0.64% (1/156) of individuals with NSHL	^25^
	0.41% (13/3,133) of individuals with NSHL	^37^
United States	0.07% (1/1,473) of general population	^27^
China	0.029% (3/10,043) of neonates	^29^
	0.25% (3/1,181) of neonates	^30^
	0.01% (8/58,397) of neonates	^31^
Japan	0% (0/1,683) of general population	This study

*HL* hearing loss, *NSHL* nonsyndromic hearing loss

Some common features of hearing loss have been identified among patients who carry the m.1555 A>G mutation. The audiometric patterns of patients with the m.1555 A>G mutation show bilateral, sensorineural, symmetric, and high-frequency hearing disorders. Hearing loss can also occur either with or without the administration of aminoglycoside antibiotics, and hearing loss can sometimes be progressive. However, hearing loss tends to be severe if aminoglycoside antibiotics have been administered^38^. In this study, the audiogram of the subject identified as a carrier of the m.1555 A>G mutation showed a bilateral, symmetric, and high-frequency hearing disorder, which corresponded to the pattern observed for mutation carriers in other studies. In Fig. 3b, we present the generation-wise average audiogram for the subjects in this study, which indicates that the hearing of the m.1555 A>G mutation carrier in this study was worse than the hearing of large numbers of noncarriers of the same generation included as subjects in this study.

Although hearing loss was relatively mild in the case under study, this subject is at a risk of progressive hearing loss if treated with aminoglycoside antibiotics. No clinically approved treatment has been reported to be effective for sensorineural hearing loss caused by the administration of aminoglycoside antibiotics; however, prescriptive, well-fitted hearing aids can improve hearing among patients with aminoglycoside-induced hearing loss^38^. Cochlear implantation has also been reported to be an effective therapeutic choice for patients with profound hearing loss caused by this mutation^38^. Hearing loss has been reported to be a significant risk factor for dementia^3^, and hearing loss is thought to have functional, social, emotional, and economic impacts. If carriers of m.1555 A>G and m.1494 C>T mutations are administered aminoglycoside antibiotics, then they may suffer from these disadvantages. Therefore, preventing the progression of hearing loss is an important goal. Usami et al. reported that they distributed drug use warning cards not only to patients but also to their relatives to improve avoidance of the administration of aminoglycoside antibiotics^14^. In our study, we performed genetic counseling and gave the participant a drug use warning card. Despite newer alternative antibiotics, aminoglycoside antibiotics are still being used worldwide^8,9^. Thus, the prevention of aminoglycoside administration to m.1555 A>G and m.1494 C>T mutation carriers is important, for example, through the distribution of drug cards.

We were able to identify one m.1555 A>G carrier among a community-dwelling population in the Iwaki district, and we provided genetic counseling and gave him a drug card advising the avoidance of aminoglycoside administration, which will likely contribute to the prevention of hearing loss progression.
