# Correction to: Prevalence of the mitochondrial 1555 A>G and 1494 C>T mutations in a community-dwelling population in Japan

**DOI:** 10.1038/s41439-020-00123-9

**Published:** 2020-10-27

**Authors:** Yasunori Maeda, Akira Sasaki, Shuya Kasai, Shinichi Goto, Shin-ya Nishio, Kaori Sawada, Itoyo Tokuda, Ken Itoh, Shin-ichi Usami, Atsushi Matsubara

**Affiliations:** 1 Department of Otorhinolaryngology, Hirosaki University Graduate School of Medicine, Hirosaki, Japan; 2 Department of Stress Response Science, Center for Advanced Medical Research, Hirosaki University Graduate School of Medicine, Hirosaki, Japan; 3 Department of Otorhinolaryngology, Shinshu University School of Medicine, Matsumoto, Japan; 4 Department of Hearing Implant Sciences, Shinshu University School of Medicine, Matsumoto, Japan; 5 Department of Social Medicine, Hirosaki University School of Medicine, Hirosaki, Japan

**Keywords:** Mitochondrial genome, Mutation, Risk factors

Correction to: *Human Genome Variation*

10.1038/s41439-020-00115-9 published online 18 September 2020

After online publication of this article, the authors noticed two errors in the “Discussion” and “Acknowledgements” section of the article.

On page 3, right side, line 24–28, Discussion;

“In this epidemiological study, the m.1555 A>G mutation was identified in 1 out of 1,683 participants from the general population of the Iwaki district (0.06%), which is slightly lower than the frequency in previous reports”.

should be

“In this epidemiological study, the m.1555 A>G mutation was identified in 1 out of 1,683 participants from the general population of the Iwaki district (0.06%), which is slightly lower than the frequency in previous reports from other countries”.

On page 5, right side, line 2, Acknowledgements;

“This work was supported by JST COI [Grant Number JPMJCE1302]”.

should be

“This work was supported by JST COI [Grant Number JPMJCE1302], Japan Agency for Medical Research and Development (16kk0205010h0001, 19kk0205023h0004), Ministry of Health, Labour and Welfare (H29-Nanchitou(Nan)-Ippan-031, 20FC1048)”.

The original article has been corrected.

The authors apologize for any inconvenience caused.

